# A national survey on COVID-19 second-wave lockdowns on older adults’ mental wellbeing, health-seeking behaviours and social outcomes across Australia

**DOI:** 10.1186/s12877-021-02352-1

**Published:** 2021-06-30

**Authors:** Joyce Siette, Karla Seaman, Laura Dodds, Kristiana Ludlow, Carly Johnco, Viviana Wuthrich, Joanne K. Earl, Piers Dawes, Paul Strutt, Johanna I. Westbrook

**Affiliations:** 1 Centre for Health Systems and Safety Research, Australian Institute of Health Innovation, Macquarie University, New South Wales 2109 Macqaurie Park, Australia; 2 Centre for Ageing, Cognition and Wellbeing, Macquarie University, New South Wales 2109 Macqaurie Park, Australia; 3 Department of Psychology, Faculty of Medicine, Health & Human Sciences, Macquarie University, New South Wales 2109 Macqaurie Park, Australia; 4 Department of Linguistics, Faculty of Medicine, Health & Human Sciences, Macquarie University, New South Wales 2109 Macqaurie Park, Australia; 5 Department of Cognitive Science, Faculty of Medicine, Health & Human Sciences, Macquarie University, New South Wales 2109 Macqaurie Park, Australia

**Keywords:** 2019 novel coronavirus diseases (COVID-19), Lifestyle restrictions, Lockdown, Wellbeing, Social networks

## Abstract

**Background:**

The impact of severe second lockdown measures on older adults’ wellbeing is unknown. We aimed to (i) identify the impact of the second lockdown that resulted from the second wave of COVID-19 cases on older Australians’ quality of life; (ii) compare the impact of second wave lockdowns in Victoria, Australia’s second most populous State, to those in other States and Territories not in lockdown.

**Methods:**

A national cross-sectional study of community-dwelling older adults completed online questionnaires for quality of life, social networks, healthcare access, and perceived impact of COVID-19 between July to September 2020. Tobit regression was used to measure the relationships of healthcare service access and social networks with quality of life of older adults in Victoria compared to those in the rest of Australia.

**Results:**

A total of 2,990 respondents (mean [SD] age, 67.3 [7.0]; 66.8 % female) participated. At time of data collection, Victoria’s second COVID-19 lockdown had been in force for an average 51.7 days. Median quality of life scores were significantly higher in Victoria compared to the rest of Australia (t_2,827_=2.25 *p* = 0.025). Being female (95 % CI, -0.051–0.020), having lower educational attainment (95 % CI, -0.089–-0.018), receiving government benefits (95 % CI, -0.054–-0.024), having small social networks (95 % CI, 0.006–0.009) and self-reported physical chronic health conditions were all independent predictors of lower quality of life.

**Conclusions:**

Longer-term studies are required to provide more robust evidence of the impact as restrictions lift and normal social conventions return.

**Supplementary Information:**

The online version contains supplementary material available at 10.1186/s12877-021-02352-1.

## Background

To contain the spread of the novel coronavirus disease (COVID-19), physical distancing (i.e., remaining at least 1.5 m from others) and stay-at-home/lockdown restrictions (requiring citizens to remain at home, unless accessing essential services) were implemented [1, 2]. Whilst lockdowns have been effective in slowing the spread of the virus, there is concern about how these infection-control measures may impact older adults’ social lives [3, 4], as they tend to be more socially isolated, have smaller networks and may also suffer from chronic illnesses and/or rely on community services [5–7].

In the early months of the pandemic, Australia was successful in containing the first wave of COVID-19 and begun to ease restrictions in May 2020 [8]. However, in Victoria, Australia’s second-most populous State (population 6.35 million), emerging clusters of community transmissions triggered the reinstatement of restrictions in an attempt to curb a second wave of COVID-19 [9]. Victoria entered its second lockdown period on 8th July when Stage 3 “Stay-at-Home” orders came into effect for residents of metropolitan Melbourne, Australia’s second biggest city, and the Mitchell Shire [10]. Citizens were not permitted to leave their homes except for shopping for food and essential items, care and caregiving, exercise, and work or study if impossible to do at home. Other Australian State and Territory Governments worked quickly to impose interstate travel and border restrictions over fears that the virus would spread across Australia, which prohibited access to States [11].

Restrictions in Melbourne were upgraded on 2nd August as authorities struggled with contact tracing and the rate of unsourced community transmission remained high [12]. More severe lockdown measures then included a curfew (8pm to 5am), travel confined to a five-kilometre radius from citizens’ homes for shopping and exercise (limited to one hour per day) and schools transitioned to online learning [12]. The rest of Victoria entered the Stage 3 lockdowns in response to the growth of cases with the Victorian Government officially declaring a “State of Disaster” which ended on 27 September [12].

Emerging research emphasises the effect of physical distancing measures on reduced psychological health and wellbeing [13, 14, 15]. The impact of reduced possibilities for socialisation on mental health and quality of life is of particularly concern for older adults. Studies show that high levels of subjective wellbeing foster physical health and longevity and that high levels of psychological wellbeing can counterbalance the negative consequences of chronic disease and disabilities [16–18], Furthermore, many older adults rely on access to social support services in their everyday lives, however, COVID-19 related restrictions prevented a range of services including paid carers, support groups and social activities in the community from operating in their usual manner [14], Despite recent studies reporting positive short-term outcomes among older adults at the population level from initial COVID-19 lockdowns, these studies may not necessarily capture the heterogeneity of outcomes of specific settings such as nursing homes or assisted living facilities and do not capture the impact of reoccurring lockdowns on their wellbeing [19].

The utilisation of healthcare services is another important consideration when investigating the impact of COVID-19 on older adults. Older adults in the US have reported cancelling medical appointments during the height of the pandemic, paralleled by a decrease in office-based primary care encounters in the US general population [13, 15, 20], Although telemedicine consults have increased [21], clinical assessments of cardiovascular risk factors such as cholesterol levels have decreased [20], Delays in treatment have been found in relation to breast cancer treatment in the US [22], imaging in Australian stroke centres [23], and missed appointments for older adults in Hong Kong [24], However, the aforementioned studies focused on the impact of COVID-19 on health utilisation in the general population[20, 22, 23] or in older primary care patients with multimorbidity [24] rather than community-dwelling older adults.

Whilst emerging research has examined the impact of COVID-19 on quality of life, social support services and health-seeking behaviours on older adults in the early months of the pandemic [13, 14, 20, 22–24], the impact of second-wave lockdowns for older adults remains largely unknown. This study aimed to (i) identify the impact of the second lockdown that resulted from the second wave of COVID-19 cases on older Australians’ quality of life; and (ii) compare the impact of second wave lockdowns in Victoria to those in other States and Territories not in lockdown.

## Methods

### Study design and setting

A national cross-sectional survey was conducted across Australia from 10 July to 28 September 2020 to coincide with the second wave of lockdown restrictions in Victoria (8 July to 27 October 2020).

### Participants

Information about the survey and a link to access the survey (online or via post) was distributed on various public platforms and social media to rapidly recruit participants from the general population. All participants had to be aged ≥ 55 years, be residing in Australia at the time of the survey and have no self-reported diagnosis of dementia. All participants provided informed written consent prior to completion of the survey. This study was approved by the Macquarie University Human Research Ethics Committee (ref 6712).

### Measures

The 45-question survey asked respondents to reflect on the last four weeks and had six parts: (i) demographics; (ii) social networks; (iii) quality of life; (iv) impact of COVID-19; (v) healthcare access; and (vi) technology use (findings are reported separately; see Supplementary Material for a copy of the questionnaire). The questionnaire asked participants to provide their age, gender, country of birth, education, and medical history.

To assess social networks, the Lubben Social Network Scale (LSNS-6) [25], a scale with robust psychometric properties and developed for use in older adults was used. It measured structural (e.g., network size), interactional (e.g., quality of exchange) and functional components (e.g., purpose of support) of the respondent’s contacts. Total scores were calculated by summing the items, with possible scores ranging from 0 to 30. Higher scores on the scale indicated better social engagement and networks.

Quality of life was measured using the EQ-5D-5L scale [26], a short, generic tool that indicated five dimensions of health-related quality of life: mobility, self-care, pain/discomfort, usual activities and anxiety/depression. For each dimension, participants rated which of the 5 levels (no problems, slight problems, moderate problems, severe problems, extreme problems) best described their current health. EQ-5D-5L data was converted into health utility scores using the time trade-off method based on the UK tariff to provide a single estimate [27], Utility scores quantify health related quality of life along a continuum that ranges from −0.59 (worst health) to 1.00 (perfect health). This scale has high discriminatory power, established convergent and known groups validity [28].

The following question was asked to better understand the overall impact of COVID-19: “Has COVID-19 had an impact on your life overall?” with three possible responses “Yes”, “No”, “Don’t know”. Participants were also asked to indicate the extent to which they agreed to COVID-19 impact statements on a 5-point Likert scale, e.g., “COVID-19 has had a positive impact on my personal relationships (e.g., with family and friends).” Healthcare access was assessed in a series of questions about utilisation of health services, including whether COVID-19 delayed treatment or affected the management of medical conditions.

### Statistical Analysis

Data were analysed in STATA V16 [29]. To identify the impact of the second lockdown on older Australians’ quality of life, categorical variables were described using percentages and continuous variables were described using means (standard deviations) and medians (interquartile ranges). One way ANOVA (parametric), Mann-Whitney (non-parametric) and Chi-square tests were used to identify whether sociodemographic characteristics, social networks, healthcare use, and impact of COVID-19, as well as quality of life, differed by States or Territories.

As the EQ-5D-5L utility scores were non-normally distributed due to a ceiling effect (Kolmogorov-Smirnov test, p < 0.05), differences between socio-demographic sub-groups were assessed using the non-parametric Mann Whitney U test (two groups) and Kruskal-Wallis one way analysis of variance (multiple groups) at the 0.0021 alpha level, following a Bonferroni adjustment for multiple testing of 24 variables (0.05/24).

To test whether any of the other differences between Victoria and the rest of Australia explained the quality of life in the Victorian sample, the Tobit regression model was used to model correlates of quality of life indexed by EQ-5D-5L. The Tobit regression model is a frequently used tool for modelling censored variables in health status measurements [30], of which a level of significance of 0.05 was used. EQ-5D-5L utility score is known as a censored variable, i.e. a large proportion of respondents have a health utility score of 1[24] and we found that responses clustered predominantly around 85 and 90 on the scale (skewness = -1.27). Based on the Biopsychosocial model [31], the impact of each of the reported biological, psychological, and social factors on quality of life was examined. This included variables such as State of residence, gender, age, area-based social disadvantage, marital status, Australia as country of birth, education level, receiving Government benefits, receiving any form of aged care services, health condition (chronic heart disease, diabetes, stroke, sight impairment, hearing impairment, COPD, high blood pressure, asthma, depression/anxiety) and social networks. Adjustment for multiple testing was not required[32]. Further sensitivity analyses were conducted for older adults aged over 65 years for the Tobit regression model and is reported in Supplementary Material.

## Results

### Participants

Participant characteristics and comparisons between Victoria and other States and Territories in Australia are described in Table 1. A total of 2,990 individuals responded to the survey, with 253 respondents from Victoria and 2,576 from the rest of Australia. At the time of data collection, COVID-19 second lockdowns had continuously been in force only in Victoria, for an average of 51.7 days (SD = 17.0). The entire sample’s mean age was 67.3 years (range 56–107) and majority of respondents were aged 55–64 years old (45.9 %). Most respondents were female (66.8 %), married (63.9 %), born in Australia (72.8 %), retired (55.9 %), and 29.8 % of respondents were of low socioeconomic status. Nearly half (42.4 %) were receiving Government benefits or pension and only 150 (5.1 %) were receiving some form of aged care service. Respondents reported a multitude of chronic health conditions. The most common condition was self-reported high blood pressure (37.4 %), followed by sight impairment (22.0 %) and depression or anxiety (20.2 %).

**Table 1:** Demographic characteristics of the sample

Variable	National (*N* = 2990) N (%)	Victoria (*N* = 257) N (%)	Rest of Australia (*N* = 2,733) N (%)	*p*-value (Group Differences)*
**State/Territory**				
NSW	2,102 (70.3)	-	-	
ACT	18 (0.6)	-	-	
VIC	257 (8.6)	-	-	
QLD	176 (5.9)	-	-	
SA	135 (4.5)	-	-	
WA	45 (1.5)	-	-	
TAS	158 (5.3)	-	-	
NT	19 (0.6)	-	-	
Unknown	80 (2.7)			
**Gender**				
Female	1,998 (66.8)	204 (79.4)	1,794 (65.6)	**< 0.001**
Male	933 (31.2)	53 (20.6)	880 (32.2)	
Unknown	59 (2.0)	0	59 (2.2)	
**Age**				
**Mean [SD]**	67.3 (7.0)	67.6 (7.2)	67.3 (7.0)	0.59
55–64	1,372 (45.9)	103 (40.1)	1,269 (46.4)	0.20
65–74	1,204 (40.3)	116 (45.1)	1,088 (39.8)	
75–84	364 (12.2)	35 (13.6)	329 (12.0)	
85+	50 (1.7)	3 (1.2)	47 (1.7)	
**SES**				
1 (Most)	320 (10.7)	11 (4.3)	309 (11.3)	**< 0.001**
2	571 (19.1)	52 (20.2)	519 (19.0)	
3	533 (17.3)	57 (22.2)	476 (17.4)	
4	476 (15.9)	68 (26.5)	408 (14.9)	
5 (least)	998 (33.4)	69 (26.8)	929 (34.0)	
Missing	95 (3.1)	-	92 (3.4)	
**Relationship status**				
Never married	208 (7.0)	185 (6.8)	23 (9.0)	0.05
Married/De facto	1,909 (63.9)	1,755 (64.2)	154 (59.9)	
Divorced/Separated but not divorced	527 (17.6)	476 (17.4)	51 (19.8)	
Widowed	285 (9.5)	256 (9.4)	29 (11.3)	
Unknown	61 (2.0)	61 (2.2)	0 (0)	
**Country of Birth**				
Australia	2,178 (72.8)	204 (79.4)	1,974 (72.2)	0.01
Other/Unknown	812 (27.2)	53 (20.6)	759 (27.8)	
**Education**				
Secondary School or less	552 (18.5)	41 (16.0)	511 (18.7)	0.02
Trade qualification	130 (4.4)	5 (2.0)	125 (4.6)	
Certificate	263 (8.8)	21 (8.2)	242 (8.9)	
Diploma	574 (19.2)	59 (23.0)	515 (18.8)	
Bachelor’s Degree	680 (22.7)	55 (21.4)	625 (22.9)	
Post-graduate degree	705 (23.6)	74 (28.8)	631 (23.1)	
Unknown	86 (2.9)	2 (0.8)	84 (3.1)	
**Retired**				
Yes	1,671 (55.9 %)	107 (41.6 %)	1,521 (55.7 %)	0.40
No	1,319 (44.1 %)	150 (58.4 %)	1,212 (44.3 %)	
**Government benefits/pension**				
Yes	1,268 (42.4)	120 (46.7)	1,148 (42.0)	0.05
No	1,635 (54.7)	135 (52.5)	1,500 (54.9)	
Unknown	87 (2.9)	2 (0.8)	85 (3.1)	
**Aged care services**				
Yes	151 (5.1)	14 (5.5)	137 (5.0)	0.15
No	2,744 (91.8)	240 (93.4)	2,504 (91.6)	
**Health status**				
Chronic heart disease	214 (7.8)	15 (5.8)	199 (7.3)	0.39
Diabetes	287 (9.6)	21 (8.2)	266 (9.7)	0.42
Stroke	73 (2.4)	5 (2.0)	68 (2.5)	0.59
Sight impairment	657 (22.0)	67 (26.1)	590 (21.6)	0.10
Hearing impairment	528 (17.7)	44 (17.1)	484 (17.7)	0.81
COPD	128 (4.3)	6 (2.3)	122 (4.5)	0.11
High blood pressure	1119 (37.4)	87 (33.9)	1,032 (37.8)	0.22
Asthma	401 (13.4)	37 (14.4)	364 (13.3)	0.63
Depression/Anxiety	604 (20.2)	60 (23.4)	544 (19.9)	0.19
**EQ-5D-5L** Mean [SD]	*N* = 2829 0.79 [0.16]	*N* = 253 0.81 [0.15]	*N* = 2576 0.79 [0.16]	0.04^a^
** Median [IQR]**	0.80 [0.72–0.88]	0.84 [0.74–0.88]	0.80 [0.72–0.88]	
Missing	161 (5.3)	4 (1.5)	156 (6.0)	
**LSNS Total** Mean [SD]	*N* = 2,841 9.8 [5.2]	*N* = 253 9.7 [5.4]	*N* = 2,588 9.8 [5.2]	0.31^b^
**LSNS Family** Mean [SD]	*N* = 2,842 4.9 [3.0]	*N* = 253 5.1 [3.0]	*N* = 2,589 4.9 [3.0]	0.51^b^
**LSNS Friends** Mean [SD]	*N* = 2,842 4.8 [3.1]	*N* = 253 4.6 [3.2]	*N* = 2,589 4.9 [3.1]	0.60^b^
**Rating of overall health during COVID-19**				
Stayed the same	2,207 (73.8)	189 (73.5)	2,018 (73.8)	0.01
Got worse	452 (15.1)	50 (19.5)	402 (14.7)	
Got better	174 (5.8)	14 (5.5)	160 (5.9)	
Missing	157 (5.3)	4 (1.7)	153 (5.6)	
**Type of COVID-19 impact**				
Positive	55 (1.8)	3 (1.8)	52 (1.9)	**< 0.001**
Negative	1,063 (35.6)	118 (45.9)	945 (34.6)	
Mix	1,265 (42.3)	113 (50.0)	1,152 (42.2)	
Missing	607 (20.3)	23 (9.0)	584 (21.4)	
**Healthcare access**				
Unable to seek medical help in the last four weeks	1,202 (40.2)	115 (44.8)	1,087 (39.8)	0.12
Delayed seeking medical help	400 (13.4)	52 (20.2)	348 (12.7)	**0.001**
Difficulty accessing healthcare services	384 (12.8)	27 (10.5)	357 (13.1)	0.24
**Healthcare Use**				
Elective hospital stay	99 (3.3)	9 (3.5)	90 (3.3)	0.86
Non-Elective hospital stay	65 (2.2)	3 (1.2)	62 (2.3)	0.25
Visited a Doctor or Nurse	1537 (51.4)	102 (39.7)	1,435 (52.5)	**< 0.001**
Visited a healthcare professional	794 (26.6)	53 (20.6)	741 (27.1)	0.02
Home visit from doctor nurse or healthcare provider	50 (1.7)	2 (0.8)	48 (1.8)	0.24
Received healthcare help at home	48 (1.6)	4 (1.6)	44 (1.6)	0.95
Participated in a tele-health consultation	845 (29.3)	100 (38.9)	745 (27.3)	**< 0.001**
Received telehealth home care help	64 (2.1)	9 (3.5)	55 (2.0)	0.02
Pharmacy	2,186 (73.1)	193 (75.1)	1,993 (72.9)	0.45
Other	393 (13.1)	34 (13.2)	359 (13.1)	0.97
**Covid-19 has had a positive impact on my personal relationships**				
Strongly Disagree	288 (9.6)	37 (14.4)	251 (9.2)	**< 0.001**
Disagree	555 (18.6)	36 (14.0)	519 (19.0)	
Neutral	1,076 (36.0)	90 (35.0)	986 (36.1)	
Agree	618 (28.7)	60 (23.4)	558 (20.4)	
Strongly agree	246 (8.2)	28 (10.9)	218 (8.0)	
Not sure	15 (0.5)	1 (0.4)	14 (0.5)	
Missing	192 (6.4)	5 (2.0)	187 (6.8)	
**Covid-19 has had a positive impact on my social relationships**				
Strongly Disagree	330 (11.0)	37 (14.4)	293 (10.7)	**< 0.001**
Disagree	833 (27.9)	72 (28.0)	761 (27.8)	
Neutral	846 (28.3)	55 (21.4)	791 (28.9)	
Agree	603 (20.2)	64 (24.9)	539 (19.7)	
Strongly agree	178 (6.0)	21 (8.2)	157 (5.7)	
Not sure	12 (0.4)	3 (1.2)	9 (0.3)	
Missing	188 (6.3)	5 (2.0)	183 (6.7)	
**Covid-19 has had a positive impact on my mental health**				
Strongly Disagree	338 (11.3)	42 (16.3)	296 (10.8)	**< 0.001**
Disagree	921 (30.8)	81 (31.5)	840 (30.7)	
Neutral	1,128 (37.7)	82 (31.9)	1,046 (38.3)	
Agree	288 (9.6)	37 (14.4)	251 (9.2)	
Strongly agree	101 (3.4)	8 (3.1)	93 (3.4)	
Not sure	19 (0.6)	2 (0.8)	17 (0.6)	
Missing	195 (6.5)	4 (2.0)	190 (7.0)	
**Covid-19 has had a positive impact on my physical health**				
Strongly Disagree	301 (10.1)	36 (14.0)	265 (9.7)	**< 0.001**
Disagree	826 (27.6)	69 (26.9)	757 (27.7)	
Neutral	993 (33.2)	68 (26.5)	925 (33.9)	
Agree	483 (16.2)	52 (20.2)	431 (15.8)	
Strongly agree	183 (6.1)	26 (10.1)	157 (5.7)	
Not sure	10 (0.3)	1 (0.4)	9 (0.3)	
Missing	194 (6.5)	5 (2.0)	189 (6.9)	
**Covid-19 has had a positive impact on my lifestyle**				
Strongly Disagree	324 (10.8)	36 (14.0)	288 (10.5)	0.005
Disagree	861 (28.8)	79 (30.7)	782 (28.6)	
Neutral	954 (31.9)	69 (26.9)	885 (32.4)	
Agree	484 (16.2)	46 (17.9)	438 (16.0)	
Strongly agree	161 (5.4)	21 (8.2)	140 (5.1)	
Not sure	12 (0.4)	1 (0.4)	11 (0.4)	
Missing	194 (6.5)	5 (2.0)	189 (6.9)	

*Level of significance of < 0.0021, highlighted in bold. Categorical tests are chi square and continuous variables are either ^a^Mann Whitney test or ^b^Oneway ANOVA test

Compared with the rest of Australia, Victorian respondents had a higher percentage of females (79.4 % vs. 65.6 %) and individuals with higher socioeconomic status (p’s < 0.001). There were no group differences in age, relationship status, country of birth, level of education, receipt of Government benefits or aged care services and health status.

### Social network

Nationally, respondents reported a mean social network score of 9.8 (range 0–24, SD = 5.17), indicating ‘at risk’ for social isolation. There were no differences in total network (9.7[SD5.2] vs. 9.8[SD5.4]), family network (5.1[SD3.0] vs. 4.9[SD3.0]) or friend network mean score (4.6[SD3.2] vs. 4.8[SD3.1]) between Victoria and the rest of Australia.

### COVID-19 impact

Most participants (79.7 %, n = 2,383) agreed that COVID-19 had an impact on their life. Of those reporting an impact, 42.3 % indicated it had both positive and negative impacts, and 35.6 % reported a negative impact only. In terms of identifying where the impact lay, 21.3–26.2 % agreed that COVID-19 had a positive impact on social relationships, physical health, and personal relationships. Over 40 % disagreed that COVID-19 had a positive impact on mental health. Compared to other Australian States or Territories, respondents in Victoria were significantly more likely to agree or strongly agree that COVID-19 positively impacted physical health (30.2 % versus 26.3 %, p < 0.001). A significantly higher proportion of Victorians reported a negative impact on mental health, when compared to the rest of Australia (47.8 % versus 40.5 %; p < 0.001).

### Healthcare access

Respondents indicated that they had often used healthcare services in the last four weeks, with visits to the pharmacy (73.1 %) being the most frequent, followed by visits to a doctor or nurse (51.4 %) or other healthcare professionals (26.6 %). Telehealth consultations constituted over a quarter of healthcare contact (29.3 %). Over half of the national sample (59.8 %) were able to seek medical help during the second lockdowns and most respondents did not delay seeking medical help (86.6 %).

Compared to the rest of Australia, respondents in Victoria reported significantly fewer doctor or nurse visits (39.7 % versus 51.4 %, *p* < 0.001) and a higher proportion of telehealth consultations (38.9 % versus 29.3 %, *p* < 0.001). A significantly higher proportion of Victorians delayed seeking medical help, compared to those in other States or Territories (20.2 % versus 12.7 %, *p* < 0.001).

### Distribution of EQ-5D-5L utility scores

**Fig. 1:**
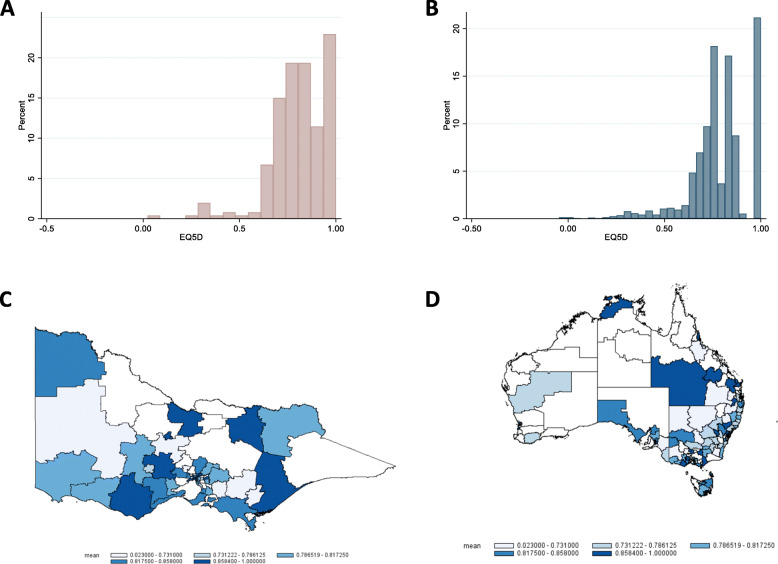
Distribution of EQ-5D-5 L utility scores as individual responses across Victoria (**A**) and Australia (**B**) and graphical representation in Victoria (**C**) and Australia (**D**). Image produced by the research team

Of all respondents, 15.1 % reported that their overall health had worsened in the last four weeks. The EQ-5D-5L utility responses were left-skewed, and responses clustered predominantly around 85 and 90 on the scale (skewness = -1.27) with 599 respondents (21.2 %) reporting no problems in any dimension for both Victoria (Fig. 1 A) and rest of Australian (Fig. 1B). Median EQ-5D-5L was 0.80 (range 0.72–0.88; mean = 0.79; SD = 0.16) in the total sample. Victorians (*n* = 253) had a median score of 0.84 (range 0.74–0.88; mean = 0.81; SD = 0.15) and those in the remaining sample (*n* = 2,576) had a median of 0.80 (range 0.72–0.88; mean = 0.79; SD = 0.16) for the rest of Australia (*p* = 0.036). A graphical distribution of EQ-5D-5L utility scores across Victoria (Fig. 1 C) and Australia (Fig. 1D) shows that quality of life scores is mixed throughout the states and territories.

**Fig. 2:**
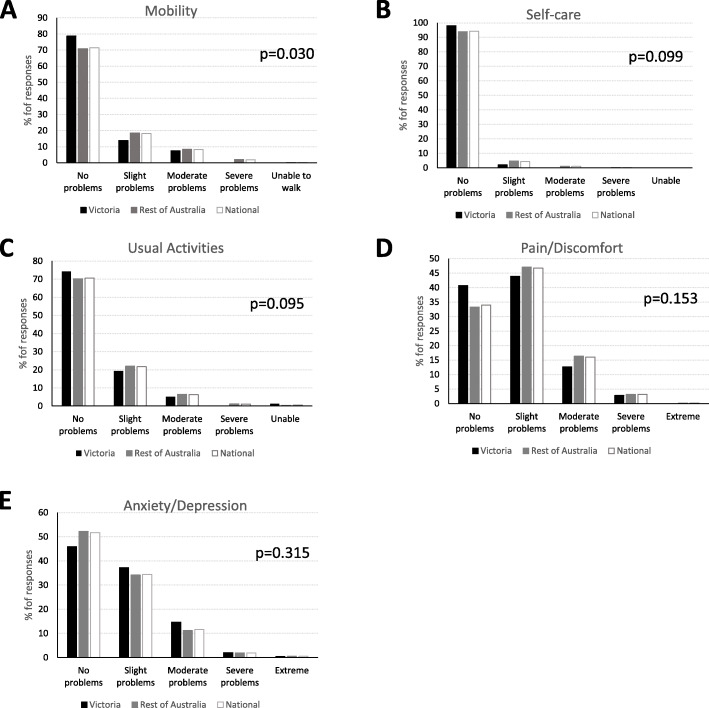
Distribution of EQ-5D-5L individual domains mobility (**A**), self-care (**B**), usual activities (**C**), pain/discomfort (**D**) and anxiety or depression (**E**)

The frequencies of item responses for each EQ-5D-5L dimension are presented in Fig. 2. In Australia, the most prevalent problems were pain and discomfort with 66.0 % reporting slight-to-extreme pain (level 2 or more), and 3.3 % reporting severe-to-extreme pain (level of 4 or 5). Respondents in Victoria had a significantly higher proportion of individuals reporting no problems for mobility compared to the other two groups (*p* = 0.03). There were no other significant group differences for the other dimensions (all *p*’s > 0.05).

### Association of EQ-5D-5L utility scores with sample characteristics

The mean EQ-5D-5L utility scores by sociodemographic, social and health service use variables for Victoria and the rest of Australia are summarised in Table 2. Lower utility scores were associated with lower socioeconomic status and those who were living alone. In the univariate analyses, there was statistically significant differences in utility scores for the whole sample in terms of different marital statuses, educational attainment, government benefits, aged care services, all nine chronic health conditions and type of COVID-19 impact (*p*’s < 0.002).

**Table 2:** EQ-5D-5L index scores for older adults during the second lockdowns

	National		*p*-value*	Victoria		Rest of Australia	
	**n**	**Median (IQR)**		**n**	**Median (IQR)**	**n**	**Median (IQR)**
**All**^**1**^	2,829	0.80 (0.72–0.88)	-	253	0.84 (0.74–0.88)	2,576	0.80 (0.72–0.88)
**Gender**			0.009				
Female	1,924	0.77 (0.71–0.88)		202	0.82 (0.74–0.88)	1,722	0.80 (0.74–0.88)
Male	905	0.84 (0.74–0.88)		51	0.84 (0.77–1)	854	0.77 (0.71–0.88)
**Age**			0.099				
55–64	1,256	0.80 (0.7–0.88)		102	0.84 (0.74–0.88)	1,154	0.80 (0.73–0.88)
65–74	1,172	0.80 (0.72–0.88)		115	0.84 (0.74–0.88)	1,057	0.80 (0.71–0.88)
75–84	354	0.80 (0.72–0.88)		33	0.84 (0.74–1)	321	0.80 (0.72–0.88)
85+	47	0.74 (0.67–0.88)		3	0.74 (0.70–1)	44	0.73 (0.65–0.88)
**SES**			**< 0.001**				
1 (Most)	307	0.77 (0.98–0.85)		11	0.77 (0.74–0.84)	296	0.77 (0.68–0.85)
2	549	0.77 (0.70–0.88)		50	0.84 (0.72–0.88)	499	0.77 (0.70–0.88)
3	517	0.77 (0.72–0.88)		57	0.77 (0.71–0.88)	460	0.77 (0.72–0.88)
4	461	0.80 (0.74–0.88)		66	0.82 (0.74–0.88)	395	0.80 (0.72–0.88)
5 (least)	966	0.84 (0.74–0.88)		69	0.84 (0.77–0.88)	897	0.84 (0.74–0.88)
Unknown	29	0.77 (0.75–0.88)		-	-	29	0.77 (0.75–0.88)
**Relationship status**			**< 0.001**				
Never married	200	0.77 (0.72–0.88)		23	0.88 (0.77–1)	177	0.77 (0.71–0.85)
Married/De facto	1,842	0.82 (0.74–0.88)		151	0.84 (0.75–0.88)	1,691	0.81 (0.74–0.88)
Divorced/ Separated but not divorced	507	0.77 (0.70–0.88)		51	0.77 (0.71–1)	456	0.77 (0.69–0.88)
Widowed	278	0.77 (0.70–0.85)		28	0.80 (0.70–0.88)	250	0.77 (0.70–0.84)
Unknown	2	0.73 (0.73–0.74)		-	-	2	0.73 (0.73–0.74)
**Country of Birth**			0.016				
Australia	2,101	0.79 (0.72–0.88)		201	0.84 (0.74–0.88)	1,900	0.77 (0.71–0.88)
Other/Unknown	728	0.84 (0.74–0.88)		52	0.83 (0.72–0.88)	679	0.84 (0.74–0.88)
**Education**			**< 0.001**				
Secondary School	521	0.77 (0.70–0.88)		40	0.84 (0.74–0.89)	481	0.77 (0.70–0.88)
Trade qualification	125	0.77 (0.67–0.84)		5	0.77 (0.74–0.84)	120	0.77 (0.67–0.84)
Certificate	258	0.77 (0.70–0.88)		21	0.84 (0.72–0.88)	237	0.77 (0.70–0.88)
Diploma	565	0.77 (0.71–0.88)		59	0.77 (0.70–0.88)	506	0.77 (0.72–0.88)
Bachelor’s Degree	667	0.80 (0.74–0.88)		54	0.78 (0.74–1)	613	0.80 (0.74–0.88)
Post-graduate degree	693	0.84 (0.74–0.88)		74	0.84 (0.77–0.88)	619	0.84 (0.74–0.88)
**Government benefits**			**< 0.001**				
Yes	1,233	0.77 (0.68–0.85)		119	0.80 (0.71–0.88)	1,114	0.77 (0.68–0.84)
No	1,595	0.84 (0.75–1)		134	0.84 (0.77–0.88)	1,461	0.84 (0.75–1)
Unknown	1	1		-	-	1	1
**Aged care services**			**< 0.001**				
Yes	148	0.69 (0.58–0.77)		14	0.72 (0.64–0.74)	134	0.68 (0.57–0.77)
No	2,677	0.80 (0.74–0.88)		238	0.84 (0.75–0.88)	2,439	0.80 (0.74–0.88)
Unknown	4	1 (0.90–1)		1	1	3	1 (0.80–1)
**Health status**							
Chronic heart disease			**< 0.001**				
Yes	209	0.77 (0.68–0.84)		15	0.77 (0.68–0.84)	194	0.77 (0.68–0.84)
No	2,620	0.80 (0.73–0.88)		238	0.84 (0.74–0.88)	2,382	0.80 (0.73–0.88)
Diabetes			**< 0.001**				
Yes	279	0.77 (0.80–0.84)		21	0.74 (0.70–0.82)	258	0.77 (0.68–0.84)
No	2,550	0.80 (0.73–0.88)		232	0.84 (0.75–0.89)	2,318	0.80 (0.73–0.88)
Stroke			**< 0.001**				
Yes	70	0.75 (0.64–0.84)		5	0.84 (0.63–0.84)	65	0.74 (0.64–0.84)
No	2,759	0.80 (0.72–0.88)		248	0.84 (0.74–0.88)	2,511	0.80 (0.72–0.88)
Sight impairment			**< 0.001**				
Yes	653	0.77 (0.69–0.84)		67	0.77 (0.71–0.85)	586	0.77 (0.69–0.84)
No	2,176	0.84 (0.74–0.88)		186	0.84 (0.74–0.91)	1,993	0.84 (0.74–0.88)
Hearing impairment			**< 0.001**				
Yes	520	0.77 (0.68–0.84)		44	0.76 (0.66–0.84)	476	0.77 (0.68–0.84)
No	2,309	0.80 (0.74–0.88)		209	0.84 (0.75–0.88)	2,100	0.80 (0.73–0.88)
COPD			**< 0.001**				
Yes	125	0.73 (0.63–0.81)		6	0.64 (0.58–0.71)	119	0.74 (0.63–0.83)
No	2,704	0.80 (0.73–0.88)		247	0.84 (0.74–0.88)	2,457	0.80 (0.73–0.88)
High blood pressure			**< 0.001**				
Yes	1,103	0.77 (0.70–0.85)		86	0.83 (0.74–0.88)	1,017	0.77 (0.70–0.84)
No	1,726	0.84 (0.74–0.88)		167	0.84 (0.74–1)	1,559	0.84 (0.74–0.88)
Asthma			**< 0.001**				
Yes	395	0.77 (0.69–0.84)		37	0.77 (0.71–0.84)	358	0.77 (0.69–0.84)
No	2,434	0.80 (0.73–0.88)		216	0.84 (0.74–0.95)	2,218	0.80 (0.73–0.88)
Depression/ Anxiety			**< 0.001**				
Yes	597	0.73 (0.64–0.77)		60	0.74 (0.65–0.82)	537	0.73 (0.64–0.77)
No	2,232	0.84 (0.74–1)		193	0.84 (0.77–1)	2,039	0.84 (0.74–1)
**Covid-19 has had a positive impact on my personal relationships**			**< 0.001**				
Strongly disagree	288	0.75 (0.65–0.84)		37	0.77 (0.66–0.84)	251	0.75 (0.65–0.84)
Disagree	555	0.77 (0.70–0.88)		36	0.82 (0.75–0.88)	519	0.77 (0.68–0.88)
Neutral	1,074	0.84 (0.74–1)		90	0.84 (0.75–1)	984	0.84 (0.74–1)
Agree	616	0.84 (0.74–0.88)		60	0.84 (0.75–0.88)	556	0.80 (0.74–0.88)
Strongly agree	246	0.84 (0.74–1)		28	0.85 (0.77–0.88)	218	0.84 (0.74–1)
Not sure	15	0.68 (0.53–0.83)		1	0.24	14	0.68 (0.53–083)
Missing	35	0.84 (0.74–0.91)		1	0.74	34	0.84 (0.74–0.91)
**Covid-19 has had a positive impact on my social relationships**			**< 0.001**				
Strongly disagree	330	0.75 (0.65–0.84)		37	0.77 (0.70–0.85)	293	0.75 (0.65–0.84
Disagree	833	0.77 (0.71–0.88)		72	0.84 (0.77–0.88)	761	0.77 (0.71–0.88)
Neutral	844	0.84 (0.75–1)		55	0.84 (0.76–1)	789	0.84 (0.74–1)
Agree	601	0.84 (0.74–0.88)		64	0.84 (0.74–0.94)	537	0.84 (0.74–0.88)
Strongly agree	178	0.77 (0.70–0.88)		21	0.77 (0.73–0.88)	157	0.77 (0.70–0.88)
Not sure	12	0.70 (0.68–0.84)		3	0.84 90.66–1)	9	0.70 (0.68–0.77)
Missing	31	0.84 (0.74–0.91)		1	0.74	30	0.84 (0.74–0.91)
**Covid-19 has had a positive impact on my mental health**			**< 0.001**				
Strongly disagree	338	0.75 (0.64–0.84)		42	0.77 (0.65–0.84)	296	0.75 (0.63–0.84)
Disagree	921	0.77 (0.70–0.88)		81	0.84 (0.75–0.88)	840	0.77 (0.69–0.88)
Neutral	1,126	0.84 (0.77–0.84)		82	0.84 (0.80–1)	1,044	0.84 (0.77–1)
Agree	286	0.80 (0.74–0.91)		37	0.77 (0.68–1)	249	0.80 (0.74–0.91)
Strongly agree	101	0.77 (0.71–0.88)		8	0.84 (0.76–0.88)	93	0.77 (0.71–0.88)
Not sure	19	0.77 (0.68–0.88)		2	0.82 (0.64–1)	17	0.77 (0.68–0.88)
Missing	38	0.82 (0.71–0.91)		1	0.74	37	0.84 (0.71–0.91)
**Covid-19 has had a positive impact on my physical health**			**< 0.001**				
Strongly disagree	301	0.75 (0.65–0.84)		36	0.77 (0.66–0.86)	265	0.75 (0.65–0.84)
Disagree	826	0.77 (0.68–0.84)		69	0.77 (0.71–0.88)	757	0.77 (0.68–0.84)
Neutral	991	0.84 (0.75–1)		68	0.84 (0.77–1)	923	0.84 (0.74–1)
Agree	481	0.84 (0.77–0.91)		52	0.84 (0.77–1)	429	0.84 (0.75–0.91)
Strongly agree	183	0.84 (0.74–1)		26	0.84 (0.68–0.88)	157	0.84 (0.75–1)
Not sure	10	0.70 (0.68–0.84)		1	1	9	0.70 (0.68–0.77)
Missing	37	0.80 (0.71–0.88)		1	0.74	36	0.82 (0.71–0.89)
**Covid-19 has had a positive impact on my lifestyle**			**< 0.001**				
Strongly disagree	324	0.75 (0.66–0.84)		36	0.76 (0.65–0.84)	288	0.75 (0.66–0.84)
Disagree	861	0.77 (0.70–0.88)		79	0.77 (0.72–0.88)	782	0.77 (0.69–0.88)
Neutral	952	0.84 (0.75–1)		69	0.84 (0.77–1)	883	0.84 (0.74–1)
Agree	482	0.84 (0.74–0.88)		46	0.85 (0.77–1)	436	0.81 (0.74–0.88)
Strongly agree	161	0.77 (0.72–0.88)		21	0.77 (0.71–0.88)	140	0.77 (0.73–0.88)
Not sure	12	0.70 (0.59–0.77)		1	0.64	11	0.70 (0.53–0.80)
Missing	37	0.84 (0.74–0.91)		1	0.74	36	0.84 (0.73–0.95)

^1^Kruskal-Wallis tests were performed. Missing data for 161 respondents; see Table 1 for more detail. *Level of Significance is *p* < 0.0021, highlighted in bold

In the final adjusted TOBIT regression model, female gender, having trade qualification and being in receipt of Government benefits or aged care services were associated with a significant negative impact on quality of life (Table 3). In addition, having higher social networks, residing in Victoria, and lack of chronic health conditions including heart disease, diabetes, hearing impairment, COPD, asthma, and depression or anxiety, were also independent, significant predictors of better health in the EQ-5D-5L. For respondents aged 65 years or more, similar findings were found. Being female, having secondary education, and receiving Government benefits or aged care services were associated with significantly lower quality of life (Supplementary material). In addition, having higher social networks and a lack of chronic health conditions (sight and hearing impairment, high blood pressure and depression) were significantly associated with better quality of life.

**Table 3:** Summary of TOBIT univariate and multivariate analyses for predictors of quality of life in 2,827 older adults

		Unadjusted			Adjusted	
	**Coefficient**	**95 % CI**	***p*-value**	**Coefficient**	**95 % CI**	***p*-value**
**Residence**						
Victoria	0.023	-0.004–0.049	0.092	0.027	0.003–0.050	**0.025**
Rest of Australia	1			1		
**Gender**						
Female	-0.026	-0.042–-0.009	**0.002**	-0.035	-0.051–-0.020	**< 0.001**
Male	1					
**Age**						
55–64	1					
65–74	-0.007	-0.024–0.009	0.382	0.007	-0.008–0.022	0.383
75–84	0.002	-0.022–0.023	0.893	0.036	0.013–0.060	**0.003**
85+	-0.059	-0.119–-0.000	0.049	-0.008	-0.062–0.046	0.765
**SES**						
1 (Most)	1					
2	0.017	-0.011–0.046	0.225	0.009	-0.016–0.034	0.489
3	0.031	0.003–0.060	0.031	0.023	-0.002–0.048	0.076
4	0.053	0.024–0.082	**< 0.001**	0.021	-0.006–0.047	0.123
5 (least)	0.063	0.037–0.089	**< 0.001**	0.021	-0.003–0.044	0.084
Unknown	0.031	-0.046–0.108	0.434	-0.013	-0.081–0.056	0.715
**Relationship status**						
Never married	1					
Married/De facto	0.030	0.001–0.060	0.045	-0.004	-0.030–0.023	0.793
Divorced/Separated but not divorced	-0.009	-0.043–0.024	0.579	-0.002	-0.032–0.027	0.890
Widowed	-0.013	-0.050–0.024	0.488	-0.008	-0.041–0.026	0.653
Unknown	-0.067	-0.345–0.210	0.634	0.008	-0.234–0.251	0.946
**Country of Birth**						
Australia	1					
Other/Unknown	0.022	0.005–0.040	0.011	0.008	-0.004–0.026	0.164
**Education**						
Secondary School or less	1					
Trade qualification	-0.036	-0.075–0.003	0.073	-0.054	-0.089–-0.018	**0.003**
Certificate	0.013	-0.018–0.043	0.415	0.009	-0.017–0.036	0.502
Diploma	0.018	-0.006–0.042	0.140	-0.002	-0.023–0.019	0.858
Bachelor’s Degree	0.042	0.019–0.066	**< 0.001**	0.009	-0.012–0.030	0.381
Post-graduate degree	0.050	0.027–0.073	**< 0.001**	0.012	-0.009–0.033	0.270
**Government benefits**						
Yes	-0.074	-0.089–-0.059	**< 0.001**	-0.039	-0.054–-0.024	**< 0.001**
No	1					
Unknown	1.096	-90.386–2.578	0.981	0.887	-41.064–42.838	0.967
**Aged care services**						
Yes	-0.180	-0.212–-0.148	**< 0.001**	-0.119	-0.150–-0.088	**< 0.001**
No	1					
Unknown	0.27	0.032–0.511	0.027	0.184	-0.038–0.407	0.104
**Health status**						
Chronic heart disease	-0.055	-0.083–-0.026	**< 0.001**	-0.023	-0.049–-0.002	**0.041**
Diabetes	-0.056	-0.081–-0.031	**< 0.001**	-0.03	-0.046–-0.001	**0.035**
Stroke	-0.097	-0.144–-0.049	**< 0.001**	-0.046	-0.088–-0.003	**0.001**
Sight impairment	-0.063	-0.080–-0.045	**< 0.001**	-0.028	-0.045–-0.012	0.066
Hearing impairment	-0.057	-0.076–-0.037	**< 0.001**	-0.017	-0.036–0.001	**< 0.001**
COPD	-0.139	-0.175–-0.104	**< 0.001**	-0.059	-0.092–-0.026	**0.022**
High blood pressure	-0.043	-0.058–-0.027	**< 0.001**	-0.016	-0.030–-0.002	0.093
Asthma	-0.057	-0.079–-0.036	**< 0.001**	-0.017	-0.036–0.003	**< 0.001**
Depression/Anxiety	-0.154	-0.171–-0.137	**< 0.001**	-0.11	-0.129–-0.095	**< 0.001**
**LSNS**	0.010	0.009–0.012	**< 0.001**	0.007	0.006–0.009	**< 0.001**

*Level of significance is < 0.05, highlighted in bold

## Discussion

We report on quality of life in a large sample in Australian older adults during second lockdowns and restrictions and its relationship to sociodemographic factors, healthcare service utilisation, social networks and attitudes towards COVID-19 impact. Results reflect a snapshot after the second severe lockdown which had been in force in Victoria for an average 51.8 days between June and September 2020.

### Impact of the second wave on older Australians

Our findings align with other investigations which show the negative effect of COVID-19 restrictions on mental wellbeing [33, 34]. The mean quality of life levels (0.79) we found during the second wave was considerably lower compared to other results from Australian studies undertaken prior to the pandemic [35]. The lower average quality of life is unsurprising and may be explained by the following factors: (1) separation from family and friends, leading to social isolation; (2) information, from multiple sources including official organisations and social media platforms, re-iterating the increased risks of COVID-19 for older adults, (3) postponements of non-critical medical appointments, and the emphasis on physical distancing, may have altered older adults’ perception of healthcare accessibility, and (4) economic and financial concerns, given the closures of businesses and termination of employment from State border lockdowns. These factors are all likely to contribute to a reduced sense of control and mastery, increased helplessness and cumulative stress and impact upon wellbeing [36]. An understanding of the relative contribution of these factors towards wellbeing would be helpful in shaping future policies and interventions; unfortunately, such data were not collected in this study.

### Impact of the second lockdown on older Victorians

Surprisingly, the mean quality of life scores of older Victorians who experienced second lockdown (0.84) was markedly higher than the mean quality of life reported by older Australians in the rest of Australia that were not in lockdown (0.80). Over 20 % of Victorian respondents reporting no problems, and there was a higher proportion of Victorians who reported no problems with their mobility compared to the rest of Australia. This better physical mobility may potentially explain the higher quality of life in the Victorian sample. However, we do not have baseline data pre-pandemic, or data during the first wave of lockdowns and it is unclear whether Victorians had better quality of life than the rest of Australia prior to the pandemic.

Despite this, it may be possible that governmental support in response to the pandemic, that is, rapid ramping-up of resources and capabilities for COVID-19 testing and provision of care, assurance that non-COVID care would not be compromised, shift towards telemedicine, and substantial stimulus packages to cushion the economic impact, might have somewhat mitigated the impact of the pandemic for Victorians. Indeed, inadequate information from health authorities can cause confusion, stress and poor quarantine adherence and can lead to increased fear, inappropriate behaviours such as stockpiling or excessive quarantine behaviours that may further escalate isolation distress [36].

In response to COVID-19 restrictions, Australia and other developed countries have seen a decrease in traditional face-to-face medical consultations and a rapid uptake of telehealth services during the pandemic. Median consultations conducted by primary health networks in Victoria increased from zero phone and 39 video consults in 2019, to over 93,000 phone and 2,500 video consults by September 2020 [21]. Our study similarly found that Victorians reported more telehealth consultations than Australians from other States (38.9 % vs. 27.3 %). Due to Government restrictions on unnecessary travel and public transport limitations, telehealth consultations may have been the only avenue in which Victorians could seek medical help. Although the Australian Government has responded with additional funded services[37] through the Medicare Benefits Schedule to enable delivery of varied telehealth services, we were not able to identify the type of telehealth appointment (e.g., counselling, supervision, psychoeducation) utilised by respondents, and therefore unable to determine exactly how telehealth could have supported wellbeing. Future studies are required to explore this connection.

Although a large proportion (40 %) of older Victorians expressed a negative impact of COVID-19 on their personal, social, lifestyle and physical behaviour, over half of the respondents reported a mix of both negative and positive impact of COVID-19. Victorians were also more divided on COVID-19 impact compared to other Australians of whom 33.7 % answered neutral (vs. 26.5 % of Victorians). Recently, common sources of joy during the lockdowns have been identified by older adults and include enjoyment with existing family and friend relationships, particularly through digital social contact, and establishment of hobbies [38]. Indeed, considering the large impact of COVID-19 on social lifestyles, digital social interactions may have been frequently used to support older adults’ wellbeing and act as a primary coping resource against loneliness [39, 40]. Furthermore, a greater percentage of Victorians in our study were more likely to report that COVID-19 had a positive and negative impact compared to the rest of Australia. For instance, 40.9 % reported that it did not have a positive impact on their physical health compared to 37.4 % of other Australians. Whilst we were unable to measure physical activity levels, maintaining a regular exercise routine is a key strategy for maintaining physical and mental health during restrictions [41, 42]. As Victorians entered second lockdowns, it may have been that they were experienced with dealing with the problems arising from home confinement and had established home workout routines [42] which enabled them to exercise.

Older adults under second lockdowns experienced further limitations on social networks and were at risk of social isolation. Our Victorian sample had unusually low mean social network scores (9.8) when compared to older Australians in the general population (15.0) [15] and older Australians receiving home care services (12.0) during the first lockdown [34]. Whilst the number of social contacts has been shown to increase following initial lockdowns [43], individuals who enter rapid subsequent lockdowns may experience more immediate, substantial and long-term impacts on social mixing patterns. Research shows that prolonged stress resulting from multiple lockdowns could lead to anxiety, depression, and the inability to manage traumatic and negative emotions, which are likely to impact on current and future social interactions [44]. Furthermore, the constant fear of contagion is likely to affect daily life and lead to further social isolation, modifying human relations in the long-run. Greater proportions of Victorians indicated that COVID-19 had negatively impacted on their mental health, personal and social relationships compared to the national sample and interventions to promote social networks of older adults may be valuable to reduce the negative social impacts of the lockdowns, and for individuals to feel re-integrated with their communities.

### Strengths and limitations

A strength of the study was the timely data collection period during COVID-19 second lockdowns in Victoria between June to September, thereby capturing the immediate impact of the second wave. Our convenience sample approach resulted in a sample which included 66% female respondents and thus was not a nationally representative sample, and therefore may not be generalisable. A further potential limitation is the cross-sectional design, which only allows comparisons of outcomes with previous literature. Future studies are required to establish a comprehensive understanding of COVID-19 on wellbeing over time.

## Conclusions

The findings from this study provide the first quantitative evidence of how second lockdowns impacted older adults and provides a basis for future comparisons of health-related quality of life as more studies in this area emerge. Overall, higher wellbeing was associated independently with residence in Victoria, male gender, better health status, and higher social networks.

## Supplementary Information

**Additional file 1.**

## Data Availability

The aggregate data that support the findings of this study are available from request from the primary author (joyce.siette@mq.edu.au).
