# Supplementary material for: A national survey on COVID-19 second-wave lockdowns on older adults’ mental wellbeing, health-seeking behaviours and social outcomes across Australia
Source: BMC Geriatr. 2021 Jun 30;21:400. doi: 10.1186/s12877-021-02352-1 (PMC8243046; doi:10.1186/s12877-021-02352-1)
Supplement: Supplementary file 1 — Additional file 1. [file 12877_2021_2352_MOESM1_ESM.docx]

**Supplementary Material**

**Supplementary Table 1. Summary of adjusted TOBIT multivariate analyses for predictors of quality of life in 1,570 adults aged over 65 years old.**

|  |  | **Adjusted** |  |
| --- | --- | --- | --- |
|  | **Coefficient** | **95% CI** | **p-value** |
| **Residence** |  |  |  |
| Victoria | 0.024 | -0.007–0.055 | 0.126 |
| Rest of Australia | 0 |  |  |
| **Gender** |  |  |  |
| Female | -0.022 | -0.043–-0.000 | **0.045** |
| Male | 0 |  |  |
| **Age** |  |  |  |
| 65-74 | 0 |  |  |
| 75-84 | 0.028 | 0.004–0.051 | **0.019** |
| 85+ | -0.014 | -0.069–0.041 | 0.627 |
| **SES** |  |  |  |
| 1 (Most) | 0 |  |  |
| 2 | 0.025 | -0.009–0.059 | 0.148 |
| 3 | 0.036 | 0.001–0.071 | **0.043** |
| 4 | 0.021 | -0.015–0.057 | 0.255 |
| 5 (least) | 0.043 | 0.011–0.075 | **0.009** |
| Unknown | -0.032 | -0.122–0.058 | 0.487 |
| **Relationship status** |  |  |  |
| Never married |  |  |  |
| Married/De facto | -0.029 | -0.068–0.011 | 0.151 |
| Divorced/Separated but not divorced | -0.019 | -0.062–0.027 | 0.890 |
| Widowed | -0.034 | -0.079–0.011 | 0.653 |
| Unknown | -0.027 | -0.273–0.220 | 0.946 |
| **Country of Birth** |  |  |  |
| Australia |  |  |  |
| Other/Unknown | 0.009 | -0.012–0.029 | 0.404 |
| **Education** |  |  |  |
| Secondary School or less |  |  |  |
| Trade qualification | -0.063 | -0.112–-0.014 | **0.003** |
| Certificate | -0.012 | -0.051–0.026 | 0.526 |
| Diploma | -0.006 | -0.035–0.022 | 0.658 |
| Bachelor’s Degree | -0.007 | -0.035–0.020 | 0.600 |
| Post-graduate degree | 0.011 | -0.027–0.029 | 0.939 |
| **Government benefits** |  |  |  |
| Yes | -0.026 | -0.046–-0.007 | **0.008** |
| No |  |  |  |
| **Aged care services** |  |  |  |
| Yes | -0.126 | -0.160–-0.092 | **<0.001** |
| No |  |  |  |
| Unknown | 0.174 | -0.049–0.397 | 0.104 |
| **Health status** |  |  |  |
| Chronic heart disease | -0.026 | -0.057–-0.006 | 0.109 |
| Diabetes | -0.016 | -0.044–0.012 | 0.270 |
| Stroke | -0.041 | -0.097–0.014 | 0.143 |
| Sight impairment | -0.037 | -0.059–-0.015 | 0.251 |
| Hearing impairment | -0.014 | -0.038–0.010 | 0.251 |
| COPD | -0.056 | -0.097–-0.014 | **0.009** |
| High blood pressure | -0.015 | -0.034–0.003 | 0.105 |
| Asthma | -0.020 | -0.049–0.008 | 0.164 |
| Depression/Anxiety | -0.109 | -0.133–-0.086 | **<0.001** |
| **LSNS** | 0.008 | 0.006–0.010 | **<0.001** |

**Level of significance is <0.05, highlighted in bold.*
